# The Role of Graph Theory in Evaluating Brain Network Alterations in Frontotemporal Dementia

**DOI:** 10.3389/fneur.2022.910054

**Published:** 2022-06-28

**Authors:** Salvatore Nigro, Marco Filardi, Benedetta Tafuri, Roberto De Blasi, Alessia Cedola, Giuseppe Gigli, Giancarlo Logroscino

**Affiliations:** ^1^Institute of Nanotechnology (NANOTEC), National Research Council, Lecce, Italy; ^2^Center for Neurodegenerative Diseases and the Aging Brain, Department of Clinical Research in Neurology, University of Bari Aldo Moro, “Pia Fondazione Cardinale G. Panico”, Tricase, Italy; ^3^Department of Basic Medicine, Neuroscience, and Sense Organs, University of Bari Aldo Moro, Bari, Italy; ^4^Department of Radiology, “Pia Fondazione Cardinale G. Panico”, Tricase, Lecce, Italy; ^5^Department of Mathematics and Physics “Ennio De Giorgi”, University of Salento, Lecce, Italy

**Keywords:** frontotemporal dementia, primary progressive aphasia, graph analysis, connectome analysis, small-world, brain networks, magnetic resonance imaging, diffusion tensor imaging

## Abstract

Frontotemporal dementia (FTD) is a spectrum of clinical syndromes that affects personality, behavior, language, and cognition. The current diagnostic criteria recognize three main clinical subtypes: the behavioral variant of FTD (bvFTD), the semantic variant of primary progressive aphasia (svPPA), and the non-fluent/agrammatic variant of PPA (nfvPPA). Patients with FTD display heterogeneous clinical and neuropsychological features that highly overlap with those presented by psychiatric syndromes and other types of dementia. Moreover, up to now there are no reliable disease biomarkers, which makes the diagnosis of FTD particularly challenging. To overcome this issue, different studies have adopted metrics derived from magnetic resonance imaging (MRI) to characterize structural and functional brain abnormalities. Within this field, a growing body of scientific literature has shown that graph theory analysis applied to MRI data displays unique potentialities in unveiling brain network abnormalities of FTD subtypes. Here, we provide a critical overview of studies that adopted graph theory to examine the topological changes of large-scale brain networks in FTD. Moreover, we also discuss the possible role of information arising from brain network organization in the diagnostic algorithm of FTD-spectrum disorders and in investigating the neural correlates of clinical symptoms and cognitive deficits experienced by patients.

## Introduction

Frontotemporal dementia (FTD) is a neurodegenerative disorder characterized by executive, behavioral, and/or language deficits (1, 2). The current diagnostic criteria recognize three main FTD subtypes according to clinical presentation: the behavioral variant of FTD (bvFTD), the semantic variant of a primary progressive aphasia (svPPA), and the non-fluent/agrammatic variant of PPA (nfvPPA) (3, 4). bvFTD is the most common subtype characterized by prominent changes in behavior and personality, as well as deficits in executive functions and social cognition (3, 5). On the other hand, loss of semantic knowledge, agrammatism, and fluency deficits are the core features of svPPA and nfvPPA (4).

The highly heterogeneous clinical and neuropsychological phenotype presented by patients with FTD makes the diagnosis of frontotemporal dementia *per se* and FTD subtypes particularly challenging, especially in the early disease stages when the symptoms are more nuanced (1). To overcome this issue several studies have used magnetic resonance imaging (MRI) to identify potential disease biomarkers and help clinicians in establishing a correct and timely diagnosis (6–8). Neuroimaging studies have consistently documented patterns of bilateral fronto-temporal gray matter alterations in patients with bvFTD (9–11). Atrophy in temporal brain regions has been associated with language impairments in patients with svPPA (7, 12), while a higher involvement of frontal regions (i.e., inferior frontal gyrus and insula) is typically observed in patients with nfvPPA (13).

More recently, several studies have applied advanced MRI acquisitions and analyses to obtain an in-depth characterization of brain alterations with respect to the simple gray matter atrophy. Particularly, an increasing number of studies have assessed brain connectivity through graph-theoretical methods, highlighting that this approach shows unique potentialities in FTD (14–29).

Graph theory is an analytical framework that allows describing the brain as a complex network identifying topological properties that reflects global and local information communication (30–33). Global and local graph properties allowed to identify specific patterns of functional and structural alteration in several neuropsychiatric and neurodegenerative disorders, including FTD subtypes (34–38). Moreover, several studies have demonstrated associations between cognitive impairments and network properties, making graph theory a suitable approach to investigate the neural correlates of cognitive performance (34). Nonetheless, graph theory results are often difficult to interpret due to the different metrics and levels (i.e., global and local) at which the analysis can be performed.

Here, we provided a step-by-step guide to interpret graph theory outcomes in FTD. Firstly, we introduced the key concepts underlying brain network construction and described the graph-based properties most frequently used to characterize topological network organization. Second, we provided a critical overview of studies that applied graph analysis in FTD by discussing functional and structural network properties and their association with clinical/neuropsychological variables. Finally, we discussed the pros and cons of graph theory approaches in FTD and points out a future research agenda.

## Graph Theory: Key Concepts and Network Construction

### Network Construction

**Figure 1:**
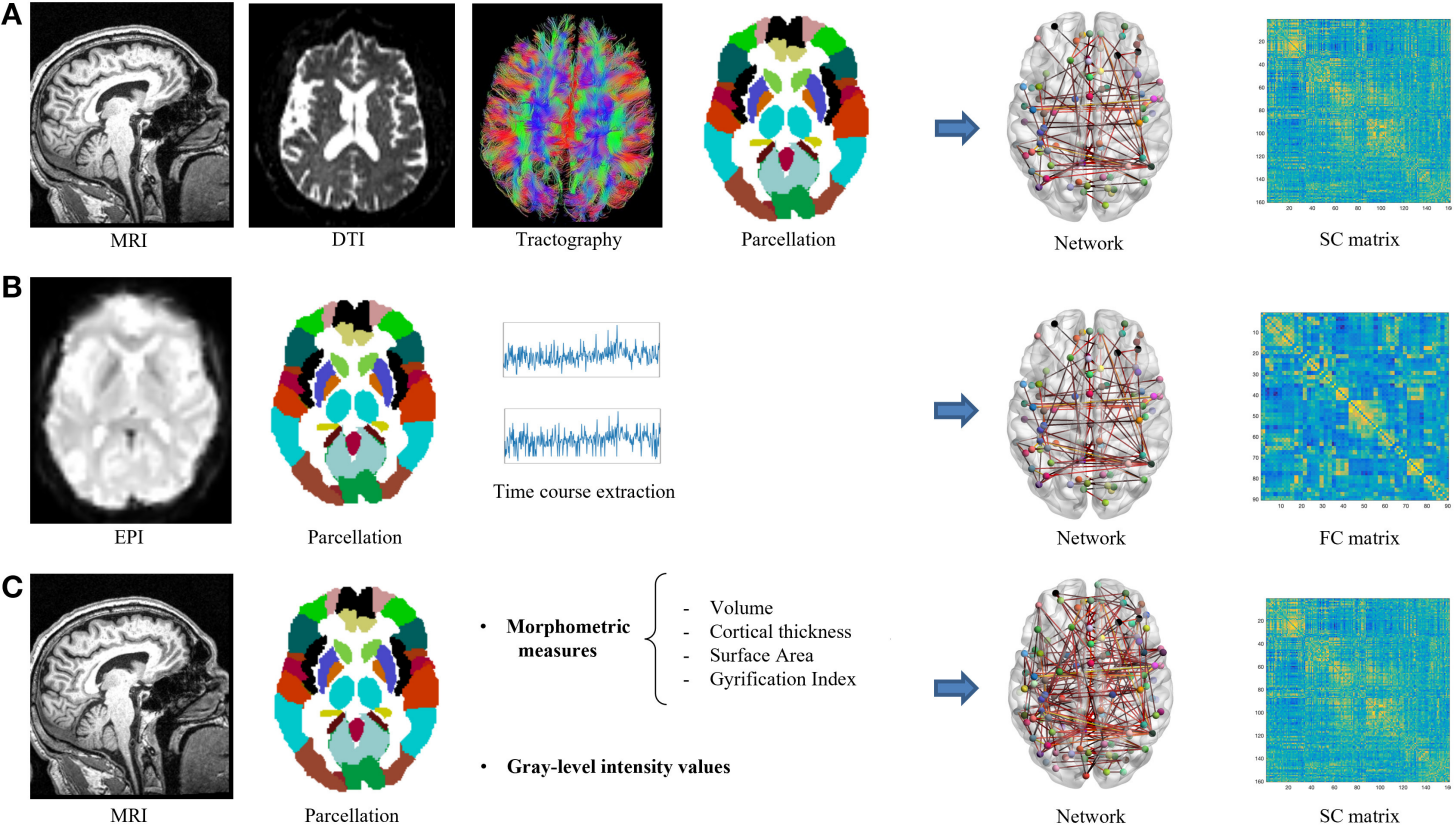
Schematic representation of brain network construction. **(A)** Diffusion tensor imaging; **(B)** resting-state fMRI; **(C)** gray matter structural covariance.

Graph theory allows modeling a network as a set of discrete elements (nodes) and their mutual relationships (edges) (30, 32, 39). Nodes usually represent predefined brain regions, and edges represent functional or structural connections between regions (30, 31). Two brain regions are considered functionally connected if they display coherent or synchronized neural activity (30, 40). Functionally connectivity is typically estimated using functional MRI (fMRI) (41), but more recent studies have shown that also single-photon emission computerized tomography (SPECT) and F-fluorodeoxyglucose positron emission tomography (FDG-PET) are reliable techniques to assess functional connections (42–44). Structural connectivity is typically estimated by the reconstruction of white matter arising from diffusion tensor imaging (DTI) (45, 46). White matter streamlines can be estimated using deterministic or probabilistic tractography, and several measures of connectivity strength (e.g., number of streamlines, fractional anisotropy, mean diffusivity) can be computed between pairs of brain regions (46, 47). The structural connectivity between brain regions can also be indirectly estimated in terms of covariation of their gray matter morphological properties (volumes, cortical thickness, surface area, and gyrification) or similarity among their gray-level intensity (48–50) based on the assumption that morphological features would covary due to shared axonal connectivity and/or genetic factors (48). For detailed information on the pros, cons, and most appropriate use of each MRI technique, we refer the readers to the study by Islam et al. (51). The defined network is represented through a connection matrix, which is typically filtered by applying thresholding and binarization approaches (52, 53). Different approaches could be used to reduce the influence of spurious connections on network topology, from the simplest application of an absolute or proportional threshold to more recent approaches such as minimum spanning tree (MST) (54). A graphical representation of the framework for the construction of a structural and functional brain network is presented in Figure 1.

### Segregation and Integration Properties

Different global and local graph metrics are used to assess features of brain network organization. Overall, they can be grouped into information processing integration and segregation metrics (30, 55, 56). Concerning brain network integration, the characteristic path length (*L*_*p*_) and global efficiency (global_E) are the most frequently used metrics (55–57). *L*_*p*_ is defined as the average shortest path length between all pairs of nodes in the network (56) and global_E is defined as the average inverse shortest path length (57). Brain networks with short *L*_*p*_ and/or high global_E are thought to transfer information across regions more efficiently (52, 56).

The modularity (*M*) and average clustering coefficient (average_Clust_C) are the two widely used metrics of brain network segregation that allow to assess information processing within specialized brain subsystems (55, 56). *M* is calculated by partitioning the network into subgroups of nodes maximizing intraconnections and minimizing interconnections (58). The average_Clust_C coefficient is defined as the average fraction in which pairs of neighboring nodes are also neighbors of each other (56). A high value of modularity and/or clustering coefficient mirror a higher propensity of the brain to execute specialized processes within interconnected brain regions (53, 56, 59). A small-world (SW) topology is characterized by high clustering and short path length, which allows to support both segregated/specialized and distributed/integrated information processing (39, 55, 57).

The above-described global metrics can also be defined at a local level to characterize integration (local path-length: local_Lp and local efficiency: local_E) and segregation (local clustering coefficient: local_Clust_C) properties for each brain region (56). Within-module degree and participation coefficient can also be computed for each node to characterize its connectivity within and across modules (58).

### Centrality Measures and Hubs Definition

Centrality measures allow to identify nodes with a high influence on the network function (56). Nodal degree (deg) is a measure of centrality defined as the number or the sum of connectivity weights of the edges incident to a node (53, 56, 59). Between centrality (*BC*) measures the fraction of shortest paths between all node pairs in the network that pass through a given index node (56, 59). Closeness centrality (*CC*) measures the mean distance between a given node and the rest of the network (30, 56, 59). Centrality measures allow the identification of network hubs, which represent topologically central regions that play a crucial role in inter-network communication (33). A brain region is usually defined as a hub when its nodal metrics are at least one standard deviation greater than the average of the corresponding measure over the entire network (21, 60). Hub regions tend to be densely interconnected and form a rich-club structure in the brain organization where the hubs are more connected among themselves than to nodes with lower centrality (33).

Regarding networks defined using the MST approach, alternative metrics are used to characterize centrality (maximum degree, maximum betweenness), distance (diameter), and topological aspects (degree divergence, leaf fraction) (54).

## Networks Alterations in Patients With FTD

Sixteen studies applied graph analysis to assess structural and functional brain network alteration in patients with FTD. Eleven studies (68.7%) compared bvFTD patients with healthy controls, one study compared svPPA patients with healthy controls, one study compared nfvPPA with healthy controls and three studies compared FTD subtypes among themselves and with healthy controls. The study from Sedeno et al. reported on a pooled sample of patients with PPA, which did not allow us to discern disease-specific information, therefore, we decided not to consider these results when discussing network alterations of PPA patients. Collectively, these studies analyzed 472 bvFTD, 70 svPPA, 94 nfvPPA, and 15 logopenic-variant primary progressive aphasia (lvPPA) patients. Detailed information for each study is reported in Table 1.

**Table 1:** Summary of studies that used graph analysis in patients with FTD.

**Reference**	**Sample**	**Mean Age**	**MMSE**	**Modality**	**Network size**	**Connectivity measures**	**Binary(B)/ weighted (W)**	**Global properties**	**Local** **properties**	**Hub (H)/ modularity (M)**
Agosta et al. (14)	50 controls 18 bvFTD	61 ± 9 61 ± 8	29 ± 1 21 ± 7	rs-fMRI	90 ROIs grouped into 8 macro-areas	Pearson's correlation	B	Clust_C, Lp global_E, Ass mean deg	deg Bc	H
Agosta et al. (15)	50 controls 13 svPPA	61.0 ± 9.0 59.4 ± 9.6	22.2 ± 7.2 29.0 ± 1.0	rs-fMRI	90 ROIs	Pearson's correlation	B	Clust_C, Lp global_E, Ass mean deg, SW	deg Bc	H
Daianu et al. (16)	37 controls 20 bvFTD 23 EOAD	59.4 ± 9.6 60.7 ± 10.7 59.6 ± 8.8	29.1 ± 0.9 24.1 ± 4.7 23.4 ± 4.2	DTI	68 ROIs	Fiber density FA MD	W	Rich club organization	deg	–
Sedeno et al. (17)	12 controls 14 bvFTD 10 stroke	62.58 ± 6.30 66.42 ± 6.83 54.50 ± 9.80	29.08 ± 1.44 25.50 ± 3.87 28.80 ± 1.09	rs-fMRI	116 ROIs grouped into 7 networks	Wavelet analysis	B	Average Bc	–	–
Sedeno et al. (18)	Site 1: 16 controls 16 bvFTD 13 FIS; Site 2: 29 controls 17 bvFTD 8 PPA; Site 3: 15 Controls 14 bvFTD 15 AD	63.50 ± 7.22 69.37 ± 7.29 62.77 ± 10.4 61.30 ± 7.16 65.23 ± 8.29 60.12 ± 5.81 69.13 ± 6.59 65.33 ± 9.12 64.07 ± 7.34	–	rs-fMRI	90 ROIs	Pearson's correlation	B/W	Lp Clust_C	deg Bc CC	–
Filippi et al. (19)	32 controls 38 bvFTD 37 EOAD	62.3 ± 2.6 63.8 ± 7.3 62.1 ± 3.9	29.3 ± 0.8 22.7 ± 5.8 19.3 ± 4.9	rs-fMRI	220 ROIs grouped into 6 macro-areas	Pearson's correlation	W	Clust_C, Lp local_E mean strength	Clust_C, Lp mean strength local_E	-
Vijverberg et al. (20)	59 bvFTD 90 AD 74 SCD	62.1 ± 6.0 63.1 ± 6.1 61.3 ± 6.6	24.6 ± 3.5 21.1 ± 5.0 28.3 ± 1.9	T1 weighted	90 ROIs	Intra-cortical similarity	B	deg, Lp Clust_C, Bc SW	deg, Lp Clust_C Bc	-
Mandelli et al. (21)	20 controls 20 nfvPPA	68.6 ± 6.0 68.8 ± 7.3	29.1 ± 1.5 26.2 ± 3.7	rs-fMRI	110 regions belonging to the speech production network	Pearson's correlation	–	global_E Lp Ass	deg Bc	H M
Reyes et al. (22)	32 controls 50 bvFTD 14 svPPA 22 nfvPPA	61.25 ± 7.28 65.85 ± 8.1 60.3 ± 7.65 63.63 ± 6.87	28.86 ± 1.27 22.47 ± 6.5 16.67 ± 7.66 16.9 ± 6.92	rs-fMRI	90 ROIs	Pearson's correlation	W	global_E Lp, deg, Clust_C, Bc	–	–
Saba et al. (23)	39 controls 41 bvFTD	61.7 ± 6.5 65.6 ± 7.01	–	rs-fMRI	116 ROIs	Wavelet correlation	B (MST)	Maximum deg, maximum Bc, diameter, Ecc, Ass, deg leaf fraction	–	–
Malpetti et al. (24)	82 controls 82 bvFTD	67.93 ± 6.95 69.37 ± 7.73	68.7 ± 1.5 71.4 ± 2.2	FDG-PET	121 ROIs	Metabolic connectivity	–	–	–	H M
Tao et al. (25)	17 controls 18 nfvPPA 15 lvPPA 9 svPPA	65 ± 8.18 69 ± 5.37 64 ± 8.12 69 ± 5.25	-	rs-fMRI	76 ROIs	Pearson's correlation	B	global_E, Lp Ass, Clust_C SW	Lp Clust_C	H
Zhou et al. (26)	20 controls 64 bvFTD	68.7 ± 1.5 71.8 ± 1.7	29.50 ± 0.1 20.08 ± 4.35	SPECT	90 ROIs	Pearson's correlation	B	global_E SW	local_E Bc deg	H
Nigro et al. (27)	20 controls 25 bvFTD	63.60 ± 5.90 66.92 ± 7.69	27.90 ± 1.68 20.80 ± 5.57	T1	82 ROIs	Joint variation	W	SW	local_E Clust_C deg	-
Ng et al. (29)	47 controls 14 bvFTD 50 AD	63.20 ± 5.00 62.05 ± 5.47 65.45 ± 5.87	29.02 ± 1.15 20.82 ± 5.66 21.21 ± 6.72	rs-fMRI	141 ROIs	Pearson's correlation	W	-	deg, local_E within-module deg partic_c	M
Nigro et al. (28)	110 controls 34 svPPA 34 nfvPPA	63.12 ± 7.49 62.91 ± 6.29 68.32 ± 7.27	29.35 ± 0.77 24.97 ± 5.10 25.54 ± 4.04	T1	82 ROIs	Joint variation	W	SW	local_E Clust_C deg	H

*bvFTD, behavioral variant of frontotemporal dementia; svPPA, semantic variant of primary progressive aphasia; nfvPPA, non-fluent/agrammatic variant of primary progressive aphasia; lvPPA, logopenic variant of primary progressive aphasia; PPA, primary progressive aphasia; EOAD, early-onset Alzheimer's disease; FIS, fronto-insular stroke; AD, Alzheimer's disease; SCD, subjective cognitive decline; MMSE, Mini-Mental State Examination; rs-fMRI, resting state functional magnetic resonance imaging; DTI, diffusion tensor imaging; FDG-PET, F-fluorodeoxyglucose positron emission tomography; SPECT, single-photon emission computed tomography; ROI, region of interest; Clust_C, clustering coefficient; Lp, path length; E, efficiency; Ass, assortativity; deg, degree; SW, small-worldness index; Bc, betweenness centrality; Ecc, eccentricity*.

### Global and Local Networks Alterations in bvFTD

Behavioral variant of FTD is by far the most extensively studied FTD dementia in terms of brain network alterations. Overall, the brain networks of patients with bvFTD showed preserved small-worldness organization, but significant alterations in global properties of the functional network have been consistently observed across studies (14, 17, 18, 23). Studies that applied graph analysis to resting state-fMRI documented alterations of both integration and segregation of information processing as reflected by lower average clustering coefficient, global efficiency, and higher characteristic path length (14, 18). A recent study that adopted MST-based analysis provided further information documenting a higher diameter and eccentricity (23), which indicates a loss of efficiency in exchange information capacity. Similar results arise from studies that applied graph theory to structural MRI (20, 27), which showed a reduced global efficiency and clustering coefficient, suggesting an overall reduced ability in information transfer. On the other hand, evidence is less conclusive for studies that assessed alterations at the local level. The majority of studies found a reduction of nodal degree, particularly evident over frontal regions (namely, orbitofrontal gyrus, anterior cingulate cortex, superior temporal pole, insula, superior and middle frontal gyri) (14, 16, 17, 19, 26), but alterations have been also observed over the left caudate nucleus, superior parietal and occipital lobes (14). A decreased integration and interconnection in temporal and frontal brain regions were also confirmed by a multicenter study investigating functional brain network organization (18). Moreover, patients with bvFTD showed an extensive reallocation of nodes across modules, most notably in the fronto-parietal, limbic-basal ganglia, and cingulum-temporal modules (24). Studies on structural MRI corroborated these findings by documenting lower local efficiency in the cortical thickness of caudal and rostral middle frontal gyrus, rostral anterior cingulate, and transverse temporal gyrus (27).

Finally, a loss of hubs over different brain regions, namely frontal gyrus (right superior frontal, inferior orbitofrontal gyri, left anterior cingulate cortex, and cuneus), basal ganglia, limbic system, cerebellum, and temporo-occipital cortex has also been reported. By contrast, new hubs appeared in the orbitofrontal and parietotemporal brain regions (14, 24).

### Global and Local Networks Alterations in svPPA

The global brain network organization of patients with svPPA was characterized by a decreased global efficiency and clustering coefficient, and a higher characteristic path length (15, 22), which could reflect lower segregation and integration in the overall network organization. This finding was also confirmed by a recent study showing a reduced small-worldness index in the structural brain network of patients (28). At a local level, a reduced nodal efficiency, degree, and clustering coefficient have been observed in several brain regions, including the left middle and superior temporal gyri, entorhinal cortex, amygdala, fusiform, hippocampus, and insula (15, 28). Moreover, a loss of hubs was observed in left-hemisphere regions (15).

### Global and Local Networks Alterations in nfvPPA

In patients with nfvPPA, a lower global efficiency was observed over the whole-brain network and in the speech production network (SPN) (21, 22). Increased path length, clustering coefficient, and modularity were also observed in the SPN (21). While the increased path length suggested a reduction in the information integration, the higher clustering coefficient and modularity may indicate a tendency of the network to segregate into smaller communities (21). At a local level, lower clustering coefficient, degree, and local efficiency were observed in several frontal regions including the left caudal and middle frontal gyrus, superior frontal gyrus, and left pars opercularis (27). Moreover, a loss of hubs in the left fronto-parietal-temporal area of the SPN, typically affected by the disease, was also documented while additional hubs were being recruited more anteriorly within the left frontal regions and in the right hemisphere (21).

### Global and Local Networks Alterations Between FTD Subtypes

When FTD subtypes were directly compared, a lower global efficiency was observed in patients with nfvPPA relative to bvFTD but not to svPPA (22). Moreover, patients with nfvPPA presented a less small-worldness index than patients with svPPA (28). At local level, significant differences were observed only between PPA subtypes. In particular, decreased clustering coefficient, degree, and local efficiency in the temporal pole were observed in patients with svPPA relative to nfvPPA. By contrast, patients with svPPA display higher values of these local metrics in the left caudal frontal gyrus and left pars opercularis than nfvPPA (28). A different configuration of hubs was also found among PPA variants (25). More in detail, both lvPPA and svPPA showed a lateralized hub distribution (right brain hemisphere) while patients with nfvPPA were characterized by a bilateral distribution across both hemispheres (25).

### Association of Brain Network Topology With Clinical/Neuropsychological

A very limited number of studies have correlated graph analysis metrics with clinical/neuropsychological impairments in FTD, with all studies specifically focused on patients with bvFTD.

A lower clustering coefficient in the right hippocampus has been associated with impairment in cognition and executive functioning, while a lower degree in the superior occipital gyrus has been associated with attentional impairments (20). Apathy and inhibition (measured through the frontal system behavior scale) showed a negative association with path length and a positive association with global efficiency, degree, and clustering (22). Increased nodal centrality in the left insular and right frontal hubs resulted associated with the degree of social cognition impairments. More recently, the severity of behavioral alterations (assessed through the neuropsychiatric inventory) was associated with lower modularity in the salience/ventral attention network and higher modularity within the module degree in the left cingulate cortex of the control network (29). Finally, higher overall cognitive functioning (assessed through the MMSE) resulted associated with higher efficiency of caudal anterior cingulate thickness (27).

## Limitations and Future Directions

The diagnosis of FTD-spectrum dementia is established based on clinical presentation, yet at the same time it is becoming increasingly reliant on neuroimaging. Indeed, the current diagnostic criteria (3, 4) require the documentation of frontal and/or anterior temporal atrophy for establishing the diagnosis of “probable” bvFTD. With the advent of new and more sophisticated analytical techniques, such as graph theory analysis and the study of connectome, neuroimaging data are likely to gain a key role in the diagnosis of dementia, including FTD subtypes. However, up to now, graph theory has been extensively applied to document altered brain connectivity in Alzheimer's disease (36, 61–63), while studies in FTD are rare and markedly skewed in favor of bvFTD, with only two studies specifically focused on svPPA and nfvPPA.

In bvFTD, graph analysis revealed a loss of efficiency in the information processing across brain regions reflected by reduced clustering coefficient and increased path length.

The pattern of neuroanatomical involvement highlighted by graph analysis overlapped with that observed in previous studies that analyzed “classic” quantitative neuroimaging metrics (i.e., gray-matter atrophy) in documenting alterations over frontal and temporal regions, further confirming their crucial role in bvFTD pathogenesis (10, 11, 64). Local network alterations showed loss of central nodes in the frontotemporal cortex and limbic system and a reorganization of network hubs, which could either mirror a compensatory process or be related to disease progression. Moreover, global and local metrics were associated with the severity of behavioral symptoms, overall cognitive functioning, and impairment in specific cognitive domains, suggesting that the alterations of information processing may exert a significant effect on the cognitive and behavioral symptoms experienced by patients.

Concerning svPPA, the few available studies documented reduced nodal efficiency, degree and clustering, and loss of hubs over several temporal and limbic regions, which indicates a reduced centrality of these regions in the information transfer. On the other hand, alterations over frontal brain regions such as the caudal middle and superior frontal gyrus were associated with nfvPPA. Moreover, patients with nfvPPA showed a reorganization of hub distribution in the speech production network and loss of hubs in the fronto–parietal–temporal areas.

When network alterations are compared between FTD subtypes, nfvPPA presented a higher impairment of global metrics compared to both bvFTD and svPPA. Moreover, svPPA and nfvPPA showed differences in local metrics: patients with nfvPPA display local abnormalities in brain regions crucial for language production (left caudal frontal gyrus and pars opercularis), while patients with svPPA showed greater impairment in areas associated with language comprehension such as the temporal pole.

Taken together, these results indicate that graph theory is capable of detecting specific brain network alterations in patients with FTD that could potentially serve as a disease biomarker. However, there is a series of methodological issues that limits its broader applicability.

First, there is a lack of standardized protocols for performing graph analysis, resulting in a wide variability of metrics and approaches across studies. Particularly the choice of thresholding, which is often arbitrary, significantly affects graph metric quantification and therefore limits the reproducibility of results. More recent techniques, such as MST, have the potential to overcome this issue but to date have been applied only in one study in the field of FTD.

Second, graph metrics are influenced by the parcellation scheme used to define network nodes, yet no consensus exists regarding which brain parcellation could be considered optimal to capture functional activity or anatomical intersubject variability. Third, all studies reviewed that analyzed fMRI focused on static functional connectivity, assuming temporal stability over scanning time. However, recent studies have reported that connectivity shows time-dependent fluctuations on the scale of seconds to minutes (65). Noteworthy, these time-dependent changes *per se* have provided novel insights into brain organization and should be considered in future studies on patients with FTD (66). Fourth, new reliable and practical frameworks need to be proposed to define graph metrics using the integration of different brain imaging modalities. Finally, all studies applied a “transversal” research design, with different graph metrics being assessed during a singular MRI session, while longitudinal studies are completely lacking, precluding the possibility to quantify the predictive value of these metrics on disease progression.

## Conclusions

Graph analysis is proven to be able to detect specific global and local brain network alterations in patients with bvFTD, while the number of studies is too limited to draw any definitive conclusions on svPPA and nfvPPA. The assessment of network alterations in FTD spectrum may have important clinical implications both in the diagnostic process, as a potential disease biomarker, and in the follow-up as an approach potentially able to track disease course.

## Author Contributions

Conceptualization: SN and GL. Data curation: BT, RDB, and AC. Investigation: SN, MF, and BT. Methodology: SN, MF, BT, RDB, and AC. Supervision: GL and GG. Writing—review and editing for important intellectual content: SN, MF, BT, AC, GG, and GL. Writing—original manuscript: SN and MF. All authors contributed to the article and approved the submitted version.

## Funding

This work has been supported with the founding of Regione Puglia and CNR for Tecnopolo per la Medicina di Precisione. D.G.R. n. 2117 of 21.11.2018 (B84I18000540002).

## Conflict of Interest

The authors declare that the research was conducted in the absence of any commercial or financial relationships that could be construed as a potential conflict of interest.

## Publisher's Note

All claims expressed in this article are solely those of the authors and do not necessarily represent those of their affiliated organizations, or those of the publisher, the editors and the reviewers. Any product that may be evaluated in this article, or claim that may be made by its manufacturer, is not guaranteed or endorsed by the publisher.
